# Tumour growth rates in squamous carcinoma of the head and neck measured by in vivo bromodeoxyuridine incorporation and flow cytometry.

**DOI:** 10.1038/bjc.1992.147

**Published:** 1992-05

**Authors:** G. Forster, T. G. Cooke, L. D. Cooke, P. D. Stanton, G. Bowie, P. M. Stell

**Affiliations:** University Department of Surgery, Glasgow Royal Infirmary, UK.

## Abstract

The cell kinetics of 82 squamous cell carcinomas of the head and neck were studied by in vivo administration of the thymidine analogue, bromodeoxyuridine (BrdUrd). Ploidy, BrdUrd labelling index (LI), duration of S-phase (Ts), potential doubling time (Tpot) and S-phase fraction (SPF) were measured by flow cytometry on 50 microns paraffin embedded sections. The range of values obtained compared well with other in vivo cell kinetic studies of head and neck cancer. Aneuploid tumours had a significantly higher BrdUrd labelling index and SPF, and a short Tpot than diploid tumours. To validate the use of 50 microns sections for measuring cell kinetic parameters by flow cytometry a comparison of values obtained by 50 microns sections and small blocks of tissue was made. No significant difference was found between the two methods. Reproducibility of values between two consecutive thick sections was also good. We conclude that reproducible cell kinetic measurements can be made in tumour samples using 50 microns sections of BrdUrd labelled tissue.


 © Macmillan Press Ltd., 1992

### Tumour growth rates in squamous carcinoma of the head and neck measured by *in vivo* bromodeoxyuridine incorporation and flow cytometry

G. Forster[1], T.G. Cooke[1], L.D. Cooke[2], P.D. Stanton[1], G. Bowie[3] & P.M. Stell[3]

[1]*University Departments of Surgery and* [2]*Otorhinolaryngology, Glasgow Royal Infirmary, Glasgow G31 2ER and* [3]*Department of Otorhinolaryngology, Royal Liverpool Hospital, Liverpool, L69 3BX, UK.*

**Summary** The cell kinetics of 82 squamous cell carcinomas of the head and neck were studied by *in vivo* administration of the thymidine analogue, bromodeoxyuridine (BrdUrd). Ploidy, BrdUrd labelling index (LI), duration of S-phase (Ts), potential doubling time (Tpot) and S-phase fraction (SPF) were measured by flow cytometry on 50 µm paraffin embedded sections. The range of values obtained compared well with other *in vivo* cell kinetic studies of head and neck cancer. Aneuploid tumours had a significantly higher BrdUrd labelling index and SPF, and a short Tpot than diploid tumours. To validate the use of 50 µm sections for measuring cell kinetic parameters by flow cytometry a comparison of values obtained by 50 µm sections and small blocks of tissue was made. No significant difference was found between the two methods. Reproducibility of values between two consecutive thick sections was also good. We conclude that reproducible cell kinetic measurements can be made in tumour samples using 50 µm sections of BrdUrd labelled tissue.

The kinetics of tumour growth may be an important factor influencing tumour aggression and the course of malignant disease. Kinetic parameters may therefore be of value in assessing prognosis and the response of tumours to cytostatic treatment (Steel, 1977; Meyer, 1982). Cell kinetic studies in man, until recently, have been limited to *in vitro* investigations because of the ethical constraints of administering radioactive DNA precursors to patients. Tritiated thymidine labelling of freshly excised tissue followed by autoradiography has been widely used (Gentili *et al.*, 1981; Brandt & Olsson, 1987; Courdi *et al.*, 1989; Meyer & Bauer, 1975). The development of flow cytometric methods for measuring DNA content, particularly in paraffin embedded archival material, has resulted in DNA ploidy and S-phase fraction analysis being performed for many tumour types (Cornelisse *et al.*, 1987; Armitage, 1985; Feichter *et al.*, 1987). Static measurements such as these provide limited information and may not give a true estimate of a tumour's proliferative state. Temporal kinetic parameters such as cell cycle phase duration and potential doubling time may provide a more meaningful estimate of tumour growth.

The potential doubling time (Steel & Bensted, 1965) is a measure of the rate of increase in the number of proliferating cells of a tumour. The equation derived by Steel takes account of the non-proliferating cells of a tumour but not cells lost from the cycling pool through death, differentiation or metastasis. Thus the Tpot represents an index of a tumour's capacity to grow and may be a clinically useful parameter. It has been suggested that radiotherapy and chemotherapy regimes can be designed on the basis of Tpot values to optimise therapeutic response for individual patients (Fowler, 1985; Thames *et al.*, 1983; Begg *et al.*, 1990). The clinical application of cell kinetic information requires a technique which generates results rapidly within a day or two of biopsy. The use of tritiated thymidine and autoradiography is therefore unsuitable.

The development of a monoclonal antibody to bromodeoxyuridine (BrdUrd) (Gratzner, 1982), a thymidine analogue which can be safely administered to patients, together with a technique that simultaneously measures incorporated bromodeoxyuridine (BrdUrd) and DNA content by flow cytometry (Dolbeare *et al.*, 1983) has facilitated the study of human tumour cell kinetics. Begg *et al.* (1985) further developed the technique to calculate the potential doubling time of tumours from a single biopsy taken a number of hours after BrdUrd administration thus obviating the need for multiple biopsies.

Here we present preliminary data on the labelling index, duration of S-phase and potential doubling times of 82 squamous cell tumours from the head and neck which form part of a prospective study to determine whether cell kinetic data, in particular the potential doubling time, have any predictive value regarding prognosis and response to treatment.

### Materials and methods

### *Selection of patients*

Patients who were due to undergo resection of a squamous cell tumour of the head and neck region were asked to participate in the study. One hundred and four patients with previously untreated and recurrent tumours from a variety of sites received BrdUrd. Permission to administer BrdUrd to patients was granted by the ethical committee at the Royal Liverpool Hospital. BrdUrd suitable for human subjects was obtained from Takeda Chemical Industries, Osaka, Japan and later from the Department of Pharmacy at the University of Strathclyde, Glasgow. The BrdUrd was administered as an intravenous infusion over 15 min at a concentration of 200 mg in 100 ml of 0.9% saline, 6-8 h before expected resection of the surgical specimen.

### *Sample preparation*

On receipt of the specimen, a sample of tumour up to 1 $cm^3$ in volume was taken close to the margin of the tumour and fixed in cold 70% alcohol. After 2-3 days half the sample was processed to paraffin wax while the remainder was left in alcohol. Thick 50 µm sections were dewaxed and rehydrated through graded alcohols to water. The sections were disaggregated according to the method described by Hedley *et al.* (1985) using 0.5% pepsin pH 1.5 at 37°C for 30 min. The digest was washed twice in phosphate buffered saline (PBS) and then filtered through a 40 µm mesh. Disaggregation of the remaining fixed specimen was performed in parallel by rehydrating to water, mincing the tumour finely as possible with a scalpel and disaggregating as above with pepsin. Thin 4 µm sections were cut from all paraffin blocks and stained with haematoxylin and eosin to confirm the presence of tumour and assess the relative proportions of tumour to normal tissue.

Correspondence: T.G. Cooke, University Department of Surgery, Queen Elizabeth Building, Glasgow Royal Infirmary, Alexandra Parade, Glasgow G31 2ER, UK.
Received 7 August 1991; and in revised form 19 December 1991.

*BrdUrd/DNA staining*

The nuclear preparation was denatured by 2 M HCl at room temperature for 30 min. 0.1 M Borax pH 8.5 was used to neutralise the acid. The samples were then washed twice, first with PBS and then with PBS containing 1% bovine serum albumin and 0.1% Tween 20 (PBT). The nuclear suspension was then incubated with 100 μl of mouse anti-BrdUrd (Dako Ltd, High Wycombe) at a dilution of 1/30 for 1 h at room temperature, washed twice in PBT, followed by incubation with 100 μl of goat anti mouse-FITC conjugated antibody (Sigma Chemical Co, Poole) for 30 min at room temperature at a dilution of 1/40. After washing thoroughly in PBT, then PBS, the samples were stained with propidium iodide at a concentration of 10 $\mu g\ ml^{-1}$ for 30 min at room temperature or at 4°C overnight.

*Flow cytometric analysis*

Flow cytometric analysis (FCM) of the samples was performed using a Coulter Epics Profile II flow cytometer fitted with a 15 mW air cooled argon ion laser emitting at 488 nm. Green and red fluorescent emissions were collected through a 525 nm band pass and 610 nm long pass filter respectively. Data acquisition was in list mode and between 10,000–100,000 nuclei were recorded. Aggregates of whole nuclei and nuclear debris were excluded from the data list by gating on the DNA area *vs* peak signal.

*Calculation of cell cycle parameters*

A typical flow cytometric output for an aneuploid tumour is illustrated in Figure 1.

The duration of S-phase (Ts) and the potential doubling time (Tpot) was calculated by the method developed by Begg *et al.* (1985). The relative movement, a measure of how far labelled cells have travelled through the S-phase in the labelling time, and the BrdUrd labelling index (LI) i.e. the number of BrdUrd labelled cells expressed as a percentage of the total cell population, were both calculated by on-screen analysis using the Epics Profile II software. When calculating the BrdUrd LI the number of labelled cells in G1 was halved to correct for the division that these cells had undergone since being labelled. For aneuploid tumours the combined LI of aneuploid and diploid cells (total LI) and that of the aneuploid component only was calculated. When calculating the total LI cells that had moved into G1 were not halved as before. A total LI was also calculated in this way for diploid tumours so that aneuploid and diploid tumours could be compared. This results in the small difference between LI and total LI for diploid tumours seen in Table II. The relative movement and the tumour LI were used to calculate values of Ts and Tpot using equations (1) and (2) below. All Tpot calculations for aneuploid tumours were made using the LI of the aneuploid component.

$$Ts = \frac{0.5}{RM-0.5} \times t \quad (1)$$

$$Tpot = \lambda \frac{Ts}{LI} \quad (2)$$

where t is the labelling time and λ is a correction factor for the non-linear age distribution of cells through the cell cycle. It was assumed to be 0.8 (Steel, 1977).

Calculation of lablling index and relative movement was often difficult for aneuploid tumours where there was overlap with a diploid population. In these cases diploid cells with labelled late S-phase and $G_2M$ nuclei overlapped with aneuploid cells labelled in the G1 and S-phase. Consequently the labelling index of the aneuploid component was artificially high and the relative movement was biased towards the aneuploid G1 causing the Ts to be longer than its 'true' value. Where there was a significant proportion of diploid cells in aneuploid tumour samples (over 80% of cases), labelled diploid cells with late S and $G_2M$ DNA content were excluded from the analysis. This was achieved by delineating the area between the end of the G1 aneuploid peak and the end of the diploid $G_2M$ peak and excluding the labelled cells in this area. Thus they made no contribution to the LI and relative movement. In aneuploid tumours where there was low labelling associated with the diploid component this procedure made little difference to the LI and the Ts calculated, but in those with high labelling the LI and Ts could be significantly reduced. The procedure was made easier when there was a clear demarcation between the labelled diploid and aneuploid cohorts of cells. S-phase fractions were measured from DNA histograms generated for BrdUrd/DNA analysis using a simple 'rectangular fit' model provided by Cytologic software, Coulter Electronics. These were corrected for background by exponential subtraction.

*Reproducibility*

Reproducibility of the method was assessed by measuring cell kinetic parameters on 12 pairs of consecutive 50 μm sections. Agreement between cell kinetic parameters measured from 50 μm sections and 0.5 $cm^3$ tissue blocks from the same tumour sample was also examined. Interobserver variation was monitored by comparing measurement of the BrdUrd labelling index made by two independent observers.

**Figure 1** Flow cytometric output of an aneuploid squamous cell tumour. **a**, DNA Histogram **b**, Bivariate cytogram of BrdUrd uptake *vs* DNA content.

**Table I** Comparison of measurements made from: (1) consecutive 50 μm sections; (2) sections *vs* tissue blocks; (3) LI by 2 independent observers, by the Bland-Altman Method; all given as ratios. Statistics describe the distribution of these ratios

| | | | *Mean ratio (95% C.I.)* | *s.d.* |
|---|---|---|---|---|
| Section 1/ | | LI | 1.05 (0.97–1.13) | 0.12 |
| Section 2 | (*n* = 12) | Ts | 1.07 (0.99–1.16) | 0.13 |
| Section/ | | LI | 1.05 (0.89–1.21) | 0.47 |
| Biopsy | (*n* = 37) | Ts | 1.09 (0.99–1.19) | 0.31 |
| Observer 1/ | | LI | 1.13 (1.07–1.19) | 0.16 |
| Observer 2 | (*n* = 37) | | | |

### *Statistical analysis*

Differences in cell cycle parameters between aneuploid and diploid tumours were compared by the Mann Whitney U test and association between SPF and BrdUrd labelling index tested by the Spearman rank correlation test. Reproducibility between consecutive 50 μm sections and agreement between cell cycle parameters measured for 50 μm sections and tissue blocks was assessed by a modification of the Bland-Altman technique, which can detect systematic error in one of the methods while allowing for variation due to tumour heterogeneity (Bland & Altman, 1986; Murray & Miller, 1990).

### Results

### *Tumour details*

One hundred and four patients received BrdUrd. One hundred and ten samples were collected, six patients having tumour at more than one site. Cell cycle parameters were successfully measured in 82 samples (75%). The main reason for failure was lack of or insufficient tumour in the sample (25 cases) followed by poor BrdUrd staining profiles (three cases). However 15 of these samples had sufficient tumour present to allow ploidy status to be determined.

### *Reproducibility*

Reproducibility data using the Bland-Altman technique are summarised in Table I. The mean ratios which ideally should be one indicate whether either set of results exceeds the other overall. The Standard Deviation (s.d.) indicates the degree of spread about the mean and thus a small figure indicates less tendency for the pairs of estimates to differ markedly.

Reproducibility between consecutive thick sections was good for both LI and Ts. This was also the case for 50 μm sections and tissue blocks with perhaps 50 μm sections producing slightly longer Ts values but this was not significant. Inter observer variation in the measurement of labelling indices was low. Calculation of the LI was performed in exactly the same way by each observer however there was a tendency for one observer (PS) to estimate the LI higher than the other (GF). The confidence interval for this difference was from 7–19% of the mean of the two estimates obtained.

### *Cell kinetic parameters*

The cell kinetic parameters for all tumours as measured on 50 μm tissue sections are summarised in Table II. Labelling indices varied from 1.2% to 30% with a median of 8.0%. The duration of S-phase ranged from 7.3 h to 37.5 h with a median of 13.7 h and the resulting potential doubling times varied from 1.2 days to 40.9 days with a median of 6.2 days.

Fifty-two tumours (63.4%) were aneuploid and 30 (36.6%) were diploid. The median labelling indices of aneuploid and diploid tumours were 9.5% and 5.0% respectively, aneuploid tumours having a significantly higher labelling index than diploid tumours (U = 730 $P<0.01$). This was still true when total labelling indices were compared where the labelling index of both tumour and normal tissue is measured. This parameter represents a fairer comparison between diploid and aneuploid tumours. The duration of S-phase was similar for both ploidy groups. The potential doubling times of aneuploid tumours were significantly shorter than diploid tumours with medians of 5.0 and 7.7 days respectively (U = 1705 $P<0.01$).

### *SPF analysis*

S-phase analysis was performed successfully on 69 tumours, 13 histograms of aneuploid tumours being unsuitable for analysis. The median SPF for all tumours was 11.5%. The SPF of aneuploid tumours was significantly higher than that of diploid tumours (U = 579, $P<0.01$), (Table II). The correlation between the BrdUrd labelling index and S-phase fraction was poor ($r = 0.67$) considering they are supposedly measuring the same parameter. SPF was on average 1.8 times greater than the BrdUrd labelling index.

### Discussion

The measurement of cell kinetic parameters *in vivo* in humans has only recently become possible using the thymidine analogue, bromodeoxyuridine (BrdUrd). The method developed by Begg *et al.* (1985) to measure LI, Ts and Tpot requires a single biopsy only, at a measured time interval after administration of BrdUrd. BrdUrd can be given several hours before a scheduled operation or biopsy with minimal disruption to normal routine and little extra workload. For these reasons *in vivo* cell kinetic studies have become a feasible alternative to *in vitro* labelling of tumour fragments. More detailed cell kinetic information such as the duration of other phases of the cell cycle and growth fraction still require multiple biopsies.

The rationale for cell kinetic studies arises from observations that the growth characteristics of a tumour may influence prognosis and the curative potential of radiotherapy and chemotherapy. High S-phase fractions and abnormal DNA content have been shown to correlate with reduced survival in a wide range of tumours (Friedlander *et al.*, 1984; Volm *et al.*, 1985) including some head and neck tumours (Holm, 1982; Franzen *et al.*, 1986) Thymidine labelling indices (TLI) have been shown to have some prognostic value, particularly

**Table II** Median and range of values of kinetic data for diploid and aneuploid tumours

| | | *All* | *Diploid* | *Aneuploid* | *Mann-Whitney (dip vs aneu)* |
|---|---|---|---|---|---|
| LI | (%) | 8.0 (1.2–30.0) | 5.0 (1.2–12.2) | 9.5 (3.7–30.0) | $P<0.01$ |
| TOTAL LI | (%) | 6.9 (1.3–21.9) | 5.6 (1.3–14.6) | 7.7 (2.3–21.9) | $P<0.01$ |
| Ts | (h) | 13.7 (7.3–37.5) | 13.5 (7.3–27.6) | 13.8 (8.0–37.5) | NS |
| Tpot | (days) | 6.2 (1.2–40.9) | 7.7 (3.1–40.9) | 5.0 (1.2–33.7) | $P<0.01$ |
| SPF | (%) | 11.5 (2.6–30.0) | 7.9 (2.6–14.7) | 14.8 (7.6–30.0) | $P<0.01$ |

in breast cancer where high TLI correlates with reduced survival (Meyer & Hixon, 1979; Gentili *et al.*, 1981) and may also predict tumour radio and chemosensitivity (Silvestrini *et al.*, 1984). The relationship between TLI and prognosis is not so well established in head and neck cancer where unlike in breast cancer the median TLI fails to divide patients into two groups with significantly different outlook (Courdi *et al.*, 1988). However Chauvel *et al.* (1989) showed in a series of head and neck tumours, that choosing a value above the median TLI as a cut-off point identified a small group of patients with significantly reduced survival when the median failed to do so.

The results obtained in this study are similar to the limited number of *in vivo* cell kinetic studies published to date. The median labelling index of 8.0% compares well with the 7.8% observed by Wilson *et al.* (1988) after *in vivo* BrdUrd labelling of nine head and neck tumours. Other *in vivo* studies employing tritiated thymidine have quoted slightly higher values ranging from 11.7–28.6% (Bresciani *et al.*, 1974; Sakuma, 1980) but the sample sizes in these studies were very small. Much larger cohorts of patients have been studied using *in vitro* thymidine labelling. Three such studies (Chauvaudra *et al.*, 1979; Silvestrini *et al.*, 1984; Chauvel *et al.*, 1989) reported a median value of 11% for head and neck tumours. Agreement between labelling indices determined from *in vitro* and *in vivo* labelling experiments is difficult to assess, but indirect evidence from simple comparisons of separate studies show the range of values are comparable. Studies specifically addressing the problem have reported good correlation between values on the whole, but with some instances of underestimation of the Li *in vitro* (Denekamp & Kallman, 1973; Chauvaudra *et al.*, 1979). Whether or not the *in vitro* LI truly reflects the actual *in vivo* labelling index, its value as a prognostic marker, at least in some tumours, is well established and still continues to be widely used.

The median Ts measured in this study of 11.5 h is similar to that previously reported for oral cancers (Sakuma, 1980; Silvestrini *et al.*, 1984). Recent *in vivo* studies have reported short mean Ts values of between 10–12 h and short median potential doubling times of 4–5 days which agree well with our values (Wilson *et al.*, 1988; Begg *et al.*, 1990).

It has been suggested that fast growing tumours may be more responsive to cytostatic therapy presumably because a large proportion of cells are in cycle. Feichter *et al.* (1987) observed that although the majority of tumours which failed to respond to therapy had a low SPF, there was a small group of non-responders with high SPF. One possible reason for this is that the cell regeneration rate is in excess of cell destruction, significant cell repopulation taking place in between treatment periods. For this reason conventional radiotherapy regimes may be unsuitable for fast growing tumours. An accelerated course of treatment where the treatment time is shortened from 4 weeks to 3 weeks or less may be more suitable in these cases (Thames *et al.*, 1983; Fowler, 1985). Such aggressive treatment presents a greater risk to the patient and may necessitate longer periods of hospitalisation. The measurement of cell cycle parameters, in particular the potential doubling time, for individual patients would identify those that may benefit from accelerated radiotherapy. Similarly, chemotherapy for patients with advanced head and neck cancer could be limited to those patients who are most likely to respond, sparing potential non-responders form debilitating chemotherapy regimes.

It has been proposed that tumours with a potential doubling time of 5 days or less may benefit from accelerated fractionation (Thames *et al.*, 1983; Fowler, 1985). Results for accelerated radiotherapy have been promising, particularly when combined with hyperfractionation (multiple fractions per day), and given over a continuous 12 day period (Continuous hyperfractionated accelerated radiotherapy – CHART) (Saunders & Dische, 1986). A multi-centre randomised trial has recently begun to test the efficacy of CHART compared with conventional treatment. It is hoped that cell kinetic data will also be available in those centres currently undertaking BrdUrd studies. Preliminary data from a similar accelerated radiotherapy trial for head and neck cancer which begun in 1986 has shown that there was improved local control of fast growing tumours as measured by *in vivo* iododeoxyuridine (IUdR) labelling when given accelerated treatment compared to conventional treatment (Begg *et al.*, 1990). Using the suggested cut-off point of 5 days to discriminate between quickly and slowly growing tumours, 28 tumours out of 82 in our study qualified as candidates for accelerated treatment. Twenty-five of these tumours were aneuploid, three were diploid. The potential doubling time of aneuploid tumours was significantly less than that of diploid tumours which may explain the observation that aneuploid tumours are often more responsive to therapy (Franzen *et al.*, 1986; Barlogie *et al.*, 1987; Guo *et al.*, 1989). We have previously reported that in advanced unresectable squamous carcinoma of the head and neck there was a significant prolongation of survival in patients with aneuploid tumours compared with patients with diploid tumours when treated with cisplatin (Cooke *et al.*, 1990). However a recent meta analysis of published data from six separate head and neck ploidy studies representing 1047 cases has cast doubt on the prognostic value of tumour DNA content in head and neck tumours other than in oral cancer (Stell, 1991).

As well as its potential use as a indicator of radio- and chemosensitivity the potential doubling time may be useful in assessing prognosis. Evidence suggests that tumours with short potential doubling times have shorter relapse free intervals and vice versa (Trott & Kummermehr, 1985). One might expect Tpot data to have greater predictive power with regard to prognosis and response to therapy than labelling indices since they represent a dynamic measure conveying more accurate information about tumour growth potential. However the superiority of Tpot over labelling index and other static measures of proliferation like SPF and ploidy remains to be proven. The discrepancy between BrdUrd LI and SPF seen in this study is consistent with other workers observations and occurs due to the presence of cells with S-phase DNA content that have failed to take up the DNA label (Allison *et al.*, 1985; Wilson *et al.*, 1985). These cells may have simply arrested in S-phase or are synthesising DNA at such a slow rate that the short exposure time of the DNA label is insufficient to allow any detectable incorporation to take place.

In this study cell kinetic measurements obtained from 50 μm sections were similar to those obtained from small blocks of tissue, and reproducibility between consecutive 50 μm sections was good thus validating their use for cell kinetic studies. The main advantage of thick sections is that only a small piece of tissue is used allowing measurements of the same or other tumour related factors to be repeated. It also provides the opportunity to separate tumour from normal stromal and inflammatory tissue such that more accurate cell kinetic measurements can be made on a predominance of tumour cells. Finally the BrdUrd LI can be measured immunohistochemically on 4 μm sections in parallel with flow cytometric measurements to determine the degree of concordance between the two methods.

The results from this pilot study indicate that cell kinetic data can be achieved in the majority of head and neck cancers, and further studies are under way to assess their clinical significance.

This work was supported by the Cancer Research Campaign.

### References

ALLISON, D.C., RIDOLPHO, P.F., ANDERSON, S. & BOSE, K. (1985). Variations in $^3$H-thymidine labelling of S-phase cells in solid mouse tumours. *Cancer Res.*, **45**, 6010.

ARMITAGE, N.C., ROBIAS, R.A., EVANS, D.F., TURNER, D.R., BALDWIN, R.W. & HARDCASTLE, J.D. (1985). The influence of tumour cell DNA abnormalities on survival in colorectal cancer. *Br. J. Surg.*, **72**, 828.

BARLOGIE, B., STASS, S., DIXON, D. & 5 others (1987). DNA aneuploidy in adult acute leukaemia. *Cancer Genet. Cytogenet.*, **28**, 213.

BEGG, A.C., MCNALLY, N.J., SHRIEVE, D.C. & KARCHER, H. (1985). A method to measure the duration of DNA synthesis and the potential doubling time from a single sample. *Cytometry*, **6**, 620.

BEGG, A.C., HOFLAND, I., MOONEN, L. & 8 others (1990). The predictive value of cell kinetic measurements in a European trial of accelerated fractionation in advanced head and neck tumours: An interim report. *Int. J. Radiat. Oncol. Biol. Phys.*, **19**, 1449.

BRANDT, L. & OLSSON, H. (1987). Survival following combination chemotherapy in advanced high grade non-Hodgkin's lymphomas. Relation to proliferative activity of the lymphoma cells. *Eur. J. Haematol.*, **38**, 437.

BLAND, J.M. & ALTMAN, D.G. (1986). Statistical methods for assessing agreement between two methods of clinical measurement. *Lancet*, **i**, 307.

BRESCIANI, F., PAOLUZI, R., BENASSI, M. & MOLL, J.L. (1974). Cell kinetics and growth of squamous cell carcinomas in man. *Cancer Res.*, **34**, 2405.

CHAUVEL, P., COURDI, A., GIOANNI, J., VALLICIONI, J., SANTINI, J. & DEMARD, F. (1989). The labelling index: a prognostic factor in head and neck carcinoma. *Radiotherapy & Oncol.*, **14**, 231.

CHAUVAUDRA, N., RICHARD, J.M. & MALAISE, E.P. (1979). Labelling index of human squamous cell carcinomas. Comparison of *in vivo* and *in vitro* methods. *Cell Tissue Kinet.*, **12**, 145.

COOKE, L.D., COOKE, T.G., BOOTZ, F. & 4 others (1990). Ploidy as a prognostic indicator in end stage squamous cell carcinoma of the head and neck region treated with cisplatinum. *Br. J. Cancer*, **61**, 759.

CORNELISSE, C.J., VAN DE VELDE, C.J.H., CASPERS, R.J.C., MOOLENAR, A.J. & HERMANS, J. (1987). DNA ploidy and survival in breast cancer patients. *Cytometry*, **8**, 225.

COURDI, A., HERY, M., CHAUVEL, P. & 6 others (1988). Prognostic value of continuous variables in breast cancer and head and neck cancer. Dependence on the cut-off level. *Br. J. Cancer*, **58**, 88.

COURDI, A., HERY, M., DAHAN, E. & 6 others (1989). Factors affecting relapse in node negative breast cancer. A multivariate analysis including the labelling index. *Eur. J. Can. Clin. Oncol.*, **25**, 351.

DENEKAMP, J. & KALLMAN, R.F. (1973). *In vitro* and *in vivo* labelling of animal tumours with tritiated thymidine. *Cell Tissue Kinet.*, **6**, 217.

DOLBEARE, F.A., GRATZNER, H.G., PALLAVICINI, M.G. & GRAY, J.W. (1983). Flow cytometric measurement of total DNA content and incorporated bromodeoxyuridine. *Proc. Natl Acad. Sci. USA.*, **80**, 5573.

FEICHTER, G.E., MAIER, H., ADLER, D., BORN, I.A., ABEL, U., HAAH, D. & GOERTTLER, K. (1987). S-phase fractions and DNA ploidy of oropharyngeal squamous epithelium carcinomas compared with histological grade, stage, response to chemotherapy and survival. *Acta Otolaryngol., (Stockh)*, **104**, 377.

FOWLER, J.F. (1985). Potential for increasing the differential response between tumours and normal tissues. Can proliferation rate be used? *Int. J. Radiat. Oncol. Biol. Phy.*, **12**, 641.

FRANZEN, G., KLINTENBERG, C., OLOFSSON, J. & RISBERG, B. (1986). DNA measurement – A review of 24 squamous cell carcinomas of the oral cavity. *Br. J. Cancer*, **53**, 643.

FRIEDLANDER, M.L., HEDLEY, D.W., TAYLOR, I.W., RUSSELL, P., COATES, A.S. & TATTERSALL, M.H.N. (1984). Influence of cellular DNA content on survival in advanced ovarian cancer. *Cancer Res.*, **44**, 397.

GENTILI, C., SANFILIPPO, O. & SILVESTRINI, R. (1981). Cell proliferation and its relationship to clinical features and relapse in breast cancers. *Cancer*, **48**, 974.

GRATZNER, H.G. (1982). Monoclonal antibody to 5 bromo and 5 iododeoxyuridine. A new reagent for detection of DNA replication. *Science*, **218**, 474.

GUO, Y.C., DESANTO, L. & OSETINSKY, G.V. (1989). Prognostic implications of nuclear DNA content in head and neck cancer. *Otolaryngol. Head Neck Surg.*, **100**, 95.

HEDLEY, D.W., FRIEDLANDER, M.L. & TAYLOR, I.W. (1985). Application of DNA flow cytometry to paraffin-embedded archival material for the study of aneuploidy and its clinical significance. *Cytometry*, **6**, 327.

HOLM, L.E. (1982). Cellular DNA amounts of squamous cell carcinoma of the head and neck region in relation to prognosis. *Laryngoscope*, **92**, 1064.

MEYER, J.S. & BAUER, W.C. (1975). *In vitro* determination of tritiated thymidine labelling index (LI). Evaluation of a method utilising hyperbaric oxygen and observations on the LI of human mammary carcinoma. *Cancer*, **36**, 1374.

MEYER, J.S. & HIXON, B. (1979). Advanced stage and early relapse of breast carcinomas associated with high thymidine labelling indices. *Cancer Res.*, **39**, 4042.

MEYER, J.S. (1982). Cell kinetic measurements of human tumours. *Human Pathol.*, **13**, 874.

MURRAY, G.D. & MILLER, R. (1990). Statistical comparison of two methods of clinical measurement. *Br. J. Surgery*, **77**, 384.

SAKUMA, J. (1980). Cell kinetics of human squamous cell carcinomas in the oral cavity. *Bull. Tokyo Med Dental Univ.*, **27**, 43.

SAUNDERS, M.I. & DISCHE, S. (1986). Radiotherapy employing three fractions in each day over a continuous period of 12 days. *Br. J. Radiol.*, **59**, 523.

SILVESTRINI, R., MOLINARI, R., COSTA, A., VOLTERRANI, F. & GARDANI, G. (1984). Short term variation in the labelling index as a predictor of radiotherapy response in human oral cavity carcinoma. *Int. J. Radiat. Oncol. Biol. Phys.*, **10**, 965.

STEEL, G.G. & BENSTED, J.P.M. (1965). *In vitro* studies of cell proliferation in tumours – I: Critical appraisal of methods and theoretical considerations. *Eur. J. Cancer*, **1**, 275.

STEEL, G.G. (1977). Growth kinetics of tumours. Oxford: Clarendon Press.

STELL, P.M. (1991). Ploidy in head and neck cancer: a review and meta analysis. *Clin. Otol.* (in press).

THAMES, H.D., PETERS, L.J., WITHERS, R. & FLETCHER, G.H. (1983). Accelerated fractionation *vs* hyperfractionation: Rationales for several treatments per day. *Int. J. Radiation Oncology Biol. Phy.*, **9**, 127.

TROTT, K.-R. & KUMMERMEHR, K. (1985). What is known about tumour proliferation rates to choose between accelerated fractionation or hyperfractionation. *Radiother. Oncol.*, **3**, 1.

VOLM, M., MATTERN, J., SONKA, J., VOGT-SCHADEN, M. & NAYSS, K. (1985). DNA distribution in non-small cell lung carcinomas and its relationship to clinical behaviour. *Cytometry*, **6**, 348.

WILSON, G.D., MCNALLY, N.J., DUNPHY, E., KRCHER, H. & PFRAGNER, R. (1985). The labelling index of human and mouse tumours assessed by bromodeoxyuridine staining *in vitro* and *in vivo* and flow cytometry. *Cytometry*, **6**, 641.

WILSON, G.D., MCNALLY, N.J., DISCHE, S., SAUNDERS, M.I., DES ROCHES, C., LEWIS, A.A. & BENNETT, M.H. (1988). Measurement of cell kinetics in human tumours *in vivo* using bromodeoxyuridine incorporation and flow cytometry. *Brit. J. Cancer*, **58**, 123.


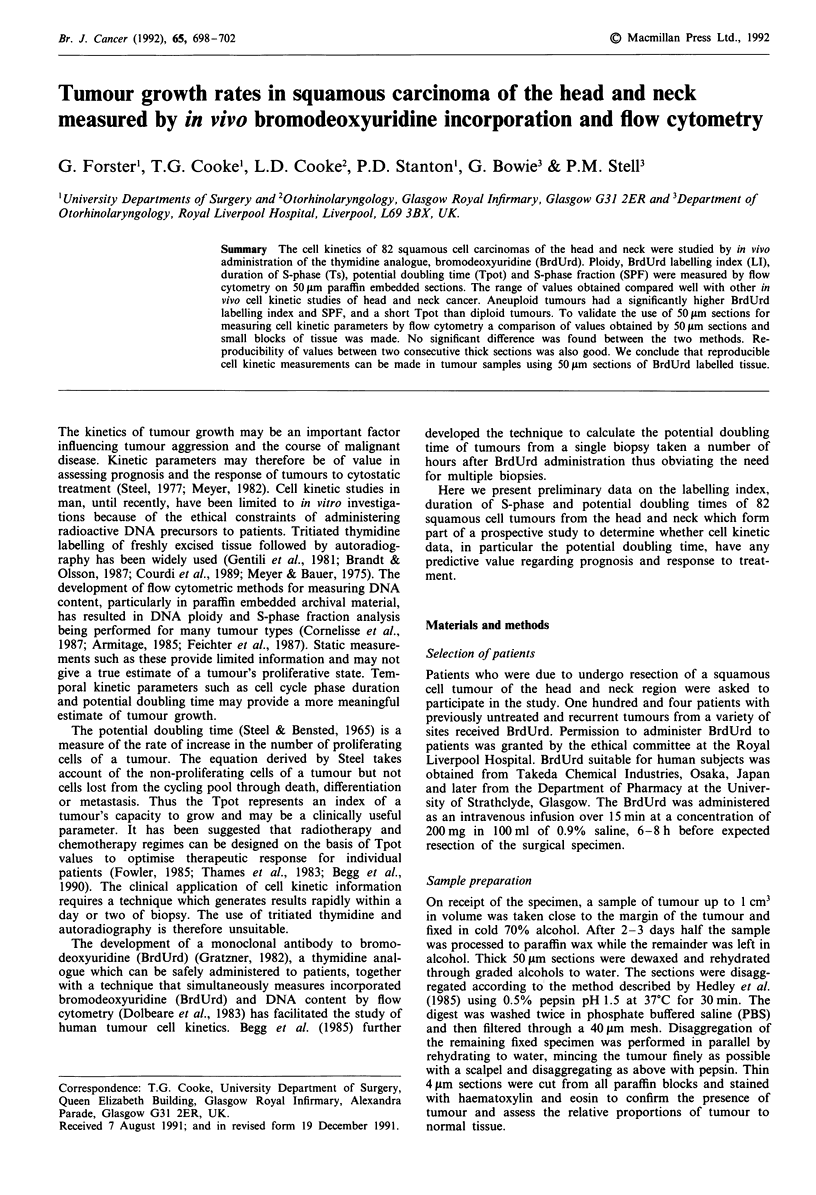

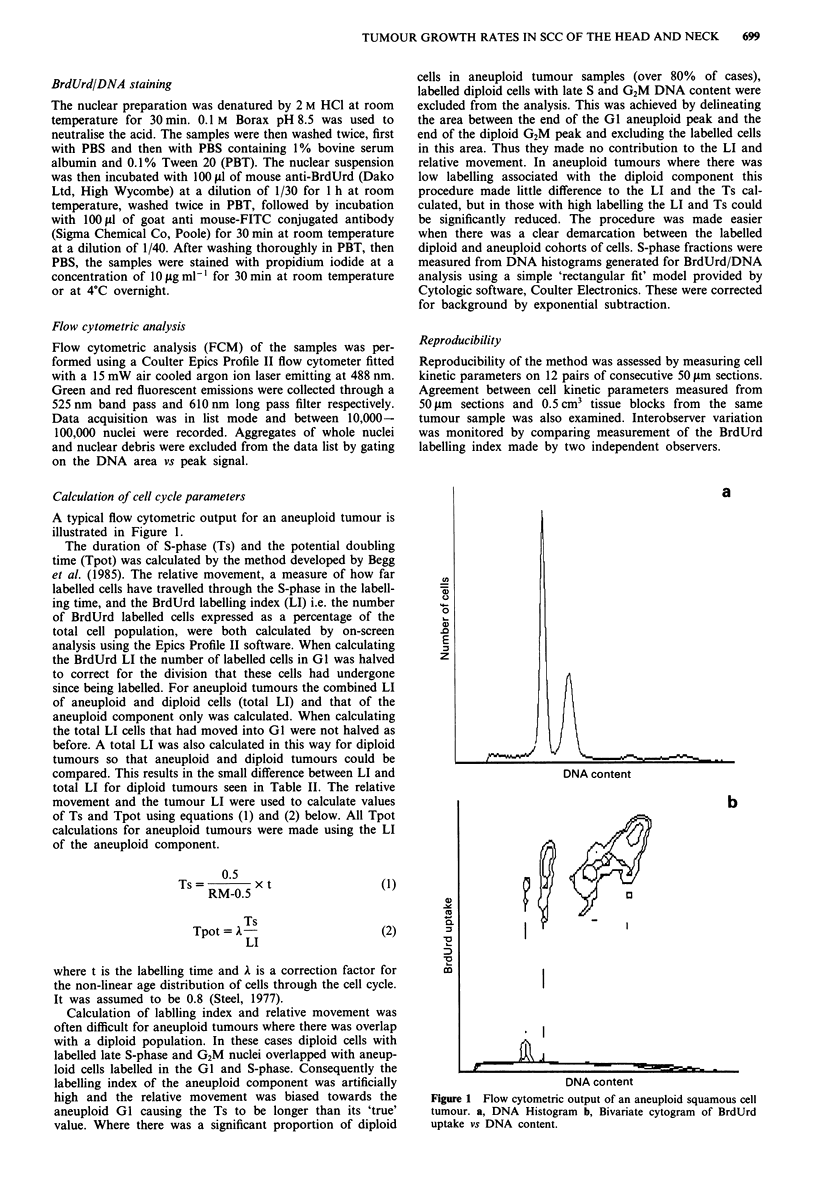

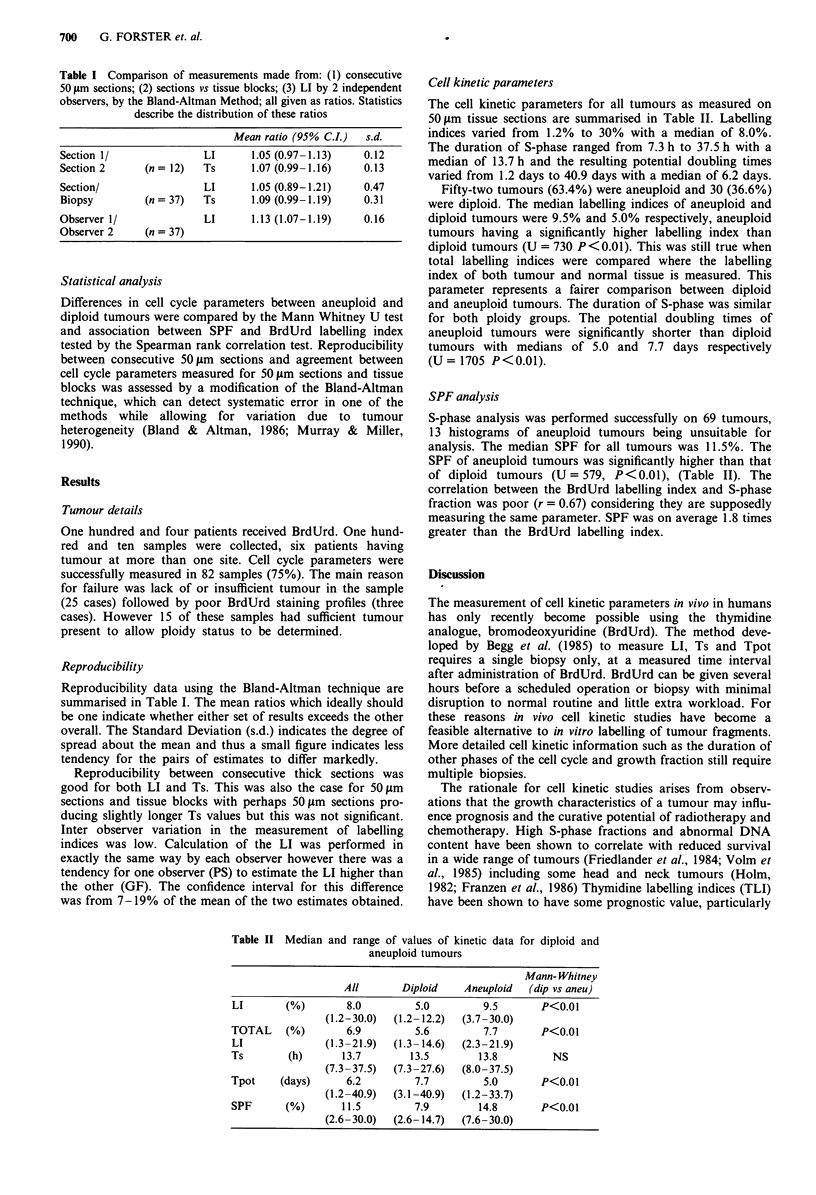

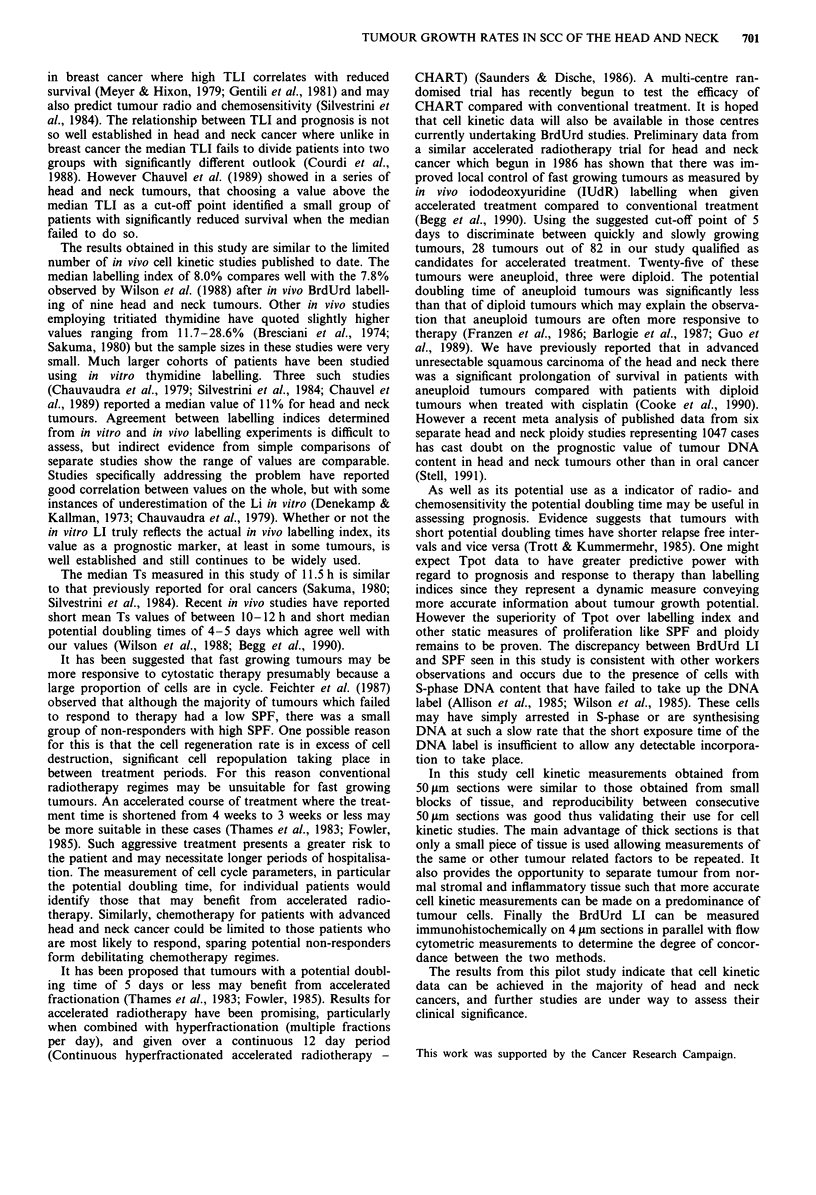

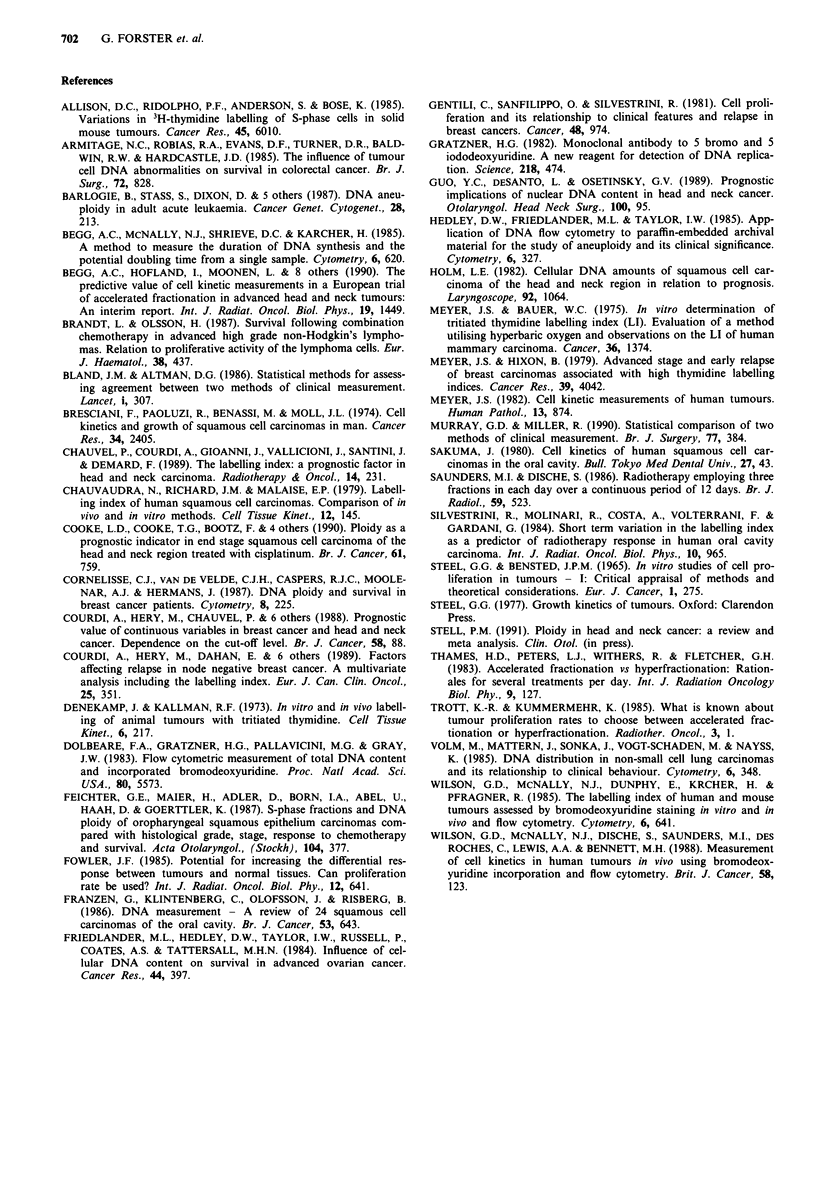


## References

[OCR_00587] Allison D. C., Ridolpho P. F., Anderson S., Bose K. (1985). Variations in the [3H]thymidine labeling of S-phase cells in solid mouse tumors.. Cancer Res.

[OCR_00594] Armitage N. C., Robins R. A., Evans D. F., Turner D. R., Baldwin R. W., Hardcastle J. D. (1985). The influence of tumour cell DNA abnormalities on survival in colorectal cancer.. Br J Surg.

[OCR_00598] Barlogie B., Stass S., Dixon D., Keating M., Cork A., Trujillo J. M., McCredie K. B., Freireich E. J. (1987). DNA aneuploidy in adult acute leukemia.. Cancer Genet Cytogenet.

[OCR_00607] Begg A. C., Hofland I., Moonen L., Bartelink H., Schraub S., Bontemps P., Le Fur R., Van Den Bogaert W., Caspers R., Van Glabbeke M. (1990). The predictive value of cell kinetic measurements in a European trial of accelerated fractionation in advanced head and neck tumors: an interim report.. Int J Radiat Oncol Biol Phys.

[OCR_00603] Begg A. C., McNally N. J., Shrieve D. C., Kärcher H. (1985). A method to measure the duration of DNA synthesis and the potential doubling time from a single sample.. Cytometry.

[OCR_00618] Bland J. M., Altman D. G. (1986). Statistical methods for assessing agreement between two methods of clinical measurement.. Lancet.

[OCR_00612] Brandt L., Olsson H. (1987). Survival following combination chemotherapy in advanced high grade non-Hodgkin's lymphomas: relation to proliferative activity of the lymphoma cells.. Eur J Haematol.

[OCR_00623] Bresciani F., Paoluzi R., Benassi M., Nervi C., Casale C., Ziparo E. (1974). Cell kinetics and growth of squamous cell carcinomas in man.. Cancer Res.

[OCR_00628] Chauvel P., Courdi A., Gioanni J., Vallicioni J., Santini J., Demard F. (1989). The labelling index: a prognostic factor in head and neck carcinoma.. Radiother Oncol.

[OCR_00633] Chavaudra N., Richard J. M., Malaise E. P. (1979). Labelling index of human squamous cell carcinomas. Comparison of in vivo and in vitro labelling methods.. Cell Tissue Kinet.

[OCR_00638] Cooke L. D., Cooke T. G., Bootz F., Forster G., Helliwell T. R., Spiller D., Stell P. M. (1990). Ploidy as a prognostic indicator in end stage squamous cell carcinoma of the head and neck region treated with cisplatinum.. Br J Cancer.

[OCR_00646] Cornelisse C. J., van de Velde C. J., Caspers R. J., Moolenaar A. J., Hermans J. (1987). DNA ploidy and survival in breast cancer patients.. Cytometry.

[OCR_00649] Courdi A., Héry M., Dahan E., Gioanni J., Abbes M., Monticelli J., Ettore F., Moll J. L., Namer M. (1989). Factors affecting relapse in node-negative breast cancer. A multivariate analysis including the labeling index.. Eur J Cancer Clin Oncol.

[OCR_00659] Denekamp J., Kallman R. F. (1973). In vitro and in vivo labelling of animal tumours with tritiated thymidine.. Cell Tissue Kinet.

[OCR_00664] Dolbeare F., Gratzner H., Pallavicini M. G., Gray J. W. (1983). Flow cytometric measurement of total DNA content and incorporated bromodeoxyuridine.. Proc Natl Acad Sci U S A.

[OCR_00670] Feichter G. E., Maier H., Adler D., Born I. A., Abel U., Haag D., Goerttler K. (1987). S-phase fractions and DNA-ploidy of oropharyngeal squamous epithelium carcinomas compared with histologic grade, stage, response to chemotherapy and survival.. Acta Otolaryngol.

[OCR_00682] Franzén G., Klintenberg C., Olofsson J., Risberg B. (1986). DNA measurement--an objective predictor of response to irradiation? A review of 24 squamous cell carcinomas of the oral cavity.. Br J Cancer.

[OCR_00687] Friedlander M. L., Hedley D. W., Taylor I. W., Russell P., Coates A. S., Tattersall M. H. (1984). Influence of cellular DNA content on survival in advanced ovarian cancer.. Cancer Res.

[OCR_00693] Gentili C., Sanfilippo O., Silvestrini R. (1981). Cell proliferation and its relationship to clinical features and relapse in breast cancers.. Cancer.

[OCR_00698] Gratzner H. G. (1982). Monoclonal antibody to 5-bromo- and 5-iododeoxyuridine: A new reagent for detection of DNA replication.. Science.

[OCR_00703] Guo Y. C., DeSanto L., Osetinsky G. V. (1989). Prognostic implications of nuclear DNA content in head and neck cancer.. Otolaryngol Head Neck Surg.

[OCR_00708] Hedley D. W., Friedlander M. L., Taylor I. W. (1985). Application of DNA flow cytometry to paraffin-embedded archival material for the study of aneuploidy and its clinical significance.. Cytometry.

[OCR_00714] Holm L. E. (1982). Cellular DNA amounts of squamous cell carcinomas of the head and neck region in relation to prognosis.. Laryngoscope.

[OCR_00719] Meyer J. S., Bauer W. C. (1975). In vitro determination of tritiated thymidine labeling index (LI). Evaluation of a method utilizing hyperbaric oxygen and observations on the LI of human mammary carcinoma.. Cancer.

[OCR_00730] Meyer J. S. (1982). Cell kinetic measurements of human tumors.. Hum Pathol.

[OCR_00725] Meyer J. S., Hixon B. (1979). Advanced stage and early relapse of breast carcinomas associated with high thymidine labeling indices.. Cancer Res.

[OCR_00738] Sakuma J. (1980). Cell kinetics of human squamous cell carcinomas in the oral cavity.. Bull Tokyo Med Dent Univ.

[OCR_00741] Saunders M. I., Dische S. (1986). Radiotherapy employing three fractions in each day over a continuous period of 12 days.. Br J Radiol.

[OCR_00746] Silvestrini R., Molinari R., Costa A., Volterrani F., Gardani G. (1984). Short-term variation in labeling index as a predictor of radiotherapy response in human oral cavity carcinoma.. Int J Radiat Oncol Biol Phys.

[OCR_00752] Steel G. G., Bensted J. P. (1965). In vitro studies of cell proliferation in tumours. I. Critical appraisal of methods and theoretical considerations.. Eur J Cancer.

[OCR_00765] Thames H. D., Peters L. J., Withers H. R., Fletcher G. H. (1983). Accelerated fractionation vs hyperfractionation: rationales for several treatments per day.. Int J Radiat Oncol Biol Phys.

[OCR_00771] Trott K. R., Kummermehr J. (1985). What is known about tumour proliferation rates to choose between accelerated fractionation or hyperfractionation?. Radiother Oncol.

[OCR_00776] Volm M., Mattern J., Sonka J., Vogt-Schaden M., Wayss K. (1985). DNA distribution in non-small-cell lung carcinomas and its relationship to clinical behavior.. Cytometry.

[OCR_00781] Wilson G. D., McNally N. J., Dunphy E., Kärcher H., Pfragner R. (1985). The labelling index of human and mouse tumours assessed by bromodeoxyuridine staining in vitro and in vivo and flow cytometry.. Cytometry.

